# A Case-Controlled Pilot Study on Rhythmic Auditory Stimulation-Assisted Gait Training and Conventional Physiotherapy in Patients With Parkinson's Disease Submitted to Deep Brain Stimulation

**DOI:** 10.3389/fneur.2020.00794

**Published:** 2020-08-04

**Authors:** Antonino Naro, Loris Pignolo, Chiara Sorbera, Desiree Latella, Luana Billeri, Alfredo Manuli, Simona Portaro, Daniele Bruschetta, Rocco Salvatore Calabrò

**Affiliations:** ^1^IRCCS Centro Neurolesi Bonino Pulejo – Piemonte, Messina, Italy; ^2^S. Anna Institute, Research in Advanced Neurorehabilitation (RAN), Crotone, Italy; ^3^Department of Biomedical, Dental Sciences and Morphological and Functional Images, University of Messina, Messina, Italy

**Keywords:** beta oscillations, deep brain stimulation (DBS), idiopathic Parkinson's disease (iPD), rhythmic auditory stimulation (RAS), treadmill gait training

## Abstract

Deep brain stimulation (DBS) is indicated when motor disturbances in patients with idiopathic Parkinson's disease (PD) are refractory to current treatment options and significantly impair quality of life. However, post–DBS rehabilitation is essential, with particular regard to gait. Rhythmic auditory stimulation (RAS)-assisted treadmill gait rehabilitation within conventional physiotherapy program plays a major role in gait recovery. We explored the effects of a monthly RAS–assisted treadmill training within a conventional physiotherapy program on gait performance and gait-related EEG dynamics (while walking on the RAS–aided treadmill) in PD patients with (*n* = 10) and without DBS (*n* = 10). Patients with DBS achieved superior results than those without DBS concerning gait velocity, overall motor performance, and the timed velocity and self-confidence in balance, sit-to-stand (and vice versa) and walking, whereas both groups improved in dynamic and static balance, overall cognitive performance, and the fear of falling. The difference in motor outcomes between the two groups was paralleled by a stronger remodulation of gait cycle–related beta oscillations in patients with DBS as compared to those without DBS. Our work suggests that RAS-assisted gait training plus conventional physiotherapy is a useful strategy to improve gait performance in PD patients with and without DBS. Interestingly, patients with DBS may benefit more from this approach owing to a more focused and dynamic re–configuration of sensorimotor network beta oscillations related to gait secondary to the association between RAS-treadmill, conventional physiotherapy, and DBS. Actually, the coupling of these approaches may help restoring a residually altered beta–band response profile despite DBS intervention, thus better tailoring the gait rehabilitation of these PD patients.

## Introduction

Deep brain stimulation (DBS) consists in the surgical implantation of needle electrodes, connected to an implantable pulse generator, in specific targets of the brain in order to manage, among other, tremor, slowness, stiffness, and walking problems caused by idiopathic Parkinson's disease (iPD) (1). DBS is particularly indicated when drug response deteriorates, the OFF periods worsen, the patient develops intolerable medication–induced dyskinesias with refractory motor fluctuations or tremor, there is not a significant improvement with regard to dopaminergic medication (<30%), and only modest improvement are appreciable during ON–state. DBS is contraindicated in patients over 75 years, with severe/malignant comorbidity considerably reducing life expectancy, chronic immunosuppression, distinct brain atrophy, and severe psychiatric disorder (2, 3).

A successful DBS allows patients to reduce their medications and improve their quality of life, given that it typically improves tremor, rigidity, bradykinesia, and levodopa-related motor complications (4, 5). Furthermore, DBS has some positive effect on gait velocity, stride length, and limbs range of movements (4, 6–8), but the overall effects on gait performance remain to be largely ascertained yet (9). Therefore, the rehabilitation plays a significant role to maintain and even potentiate motor outcomes, to counterbalance any functional impairment caused by or associated with DBS, and to adjust further DBS and medication for the rehabilitation therapy (10–12).

Conventional physical therapy and treadmill-assisted gait training provide patients with PD with great improvements in balance and gait stability and velocity (13–16). However, a few studies have assessed gait in PD patients who underwent DBS, reporting some promising results (4, 5, 9, 10). Integrating treadmill-assisted gait training equipped with rhythmic auditory stimulation (RAS) with physiotherapy has been shown to increase further speed, cadence, stride length, gait symmetry and stability in PD patients, because RAS works as a peripheral timekeeper (17–19). However, patients with DBS have not been assessed specifically for physiotherapy plus RAS-treadmill gait training aftereffects yet (14, 20, 21). Furthermore, the possible interactions between physiotherapy plus RAS-treadmill and DBS aftereffects on the neural oscillations related to gait cycle have not been investigated so far. Given that physiotherapy, RAS-treadmill, and DBS largely affects the pathological beta band activity in the corticothalamic-basal ganglia network (20, 22), a possible functional correlation between their effects on beta oscillations and gait could be hypothesized and potentially harnessed to maximize functional outcome recovery in patients with PD. Actually, physiotherapy plus RAS-treadmill could reveal and potentially restore a residually altered beta-band response profile despite DBS intervention. The prominent involvement of beta oscillatory activity is not surprising, as this is in keeping with its role in motor control and PD pathophysiology. Actually, beta regulation is a feature of motor control (including high levels during tonic contractions and low levels during dynamic voluntary movement) throughout the central nervous system and motor units (23, 24), and beta oscillations are increased in the basal ganglia and cortex in PD patients, being considered as a marker of the parkinsonian state (25–28).

To the best of our knowledge, no study evaluated such a putative functional correlation in a rehab perspective hitherto. Our study was aimed at assessing the motor and cognitive aftereffects of RAS-assisted treadmill training within a conventional rehabilitation program in PD patients with and without DBS. Furthermore, we proved whether DBS may interact with RAS-treadmill and physiotherapy according to scalp EEG data. In this regard, we explored the differences in EEG signatures between PD patients with and without DBS provided with an intensive, conventional and RAS–assisted treadmill gait training. This information may be useful to plan larger studies aimed at better tailoring gait rehabilitation in these PD patients.

## Materials and Methods

### Study Population

In this pilot study, we consecutively screened all patients with a diagnosis of iPD who were hospitalized at our Institute between January and December 2019 for intensive rehabilitation training. Patients had to be bilateral subthalamic nucleus (STN) DBS user from at least 12 months prior to study inclusion (namely, DBS group), scored between 2 and 3 at the Hoehn–Yahr modified scale (H&Y) (being able to walk at least 10 meters without assistance), and scored at least 25 on the Mini Mental State Examination (MMSE). Furthermore, they had to be stable concerning medications dose with DBS, and without adequate response to treatment and with complications related to prolonged use of levodopa prior to DBS surgery. Patients who underwent treadmill training and/or other particular physiotherapy interventions in the previous 6 months, with other neurological, orthopedic, severe visual and auditory disorders, and taking other drugs acting on the central nervous system or modulating EEG dynamics than anti-parkinsonian drugs were excluded from the study. Fifteen patients matched the inclusion criteria, but only 10 were included in the study (three refused, two had problems with the DBS device). The enrolled patients were compared with a control group of 10 PD individuals do not using DBS, matched for age, gender, disease duration, H&Y stage, Levodopa equivalent daily dosage (LEDD), and MMSE (non–DBS group). Given the exploratory nature of the present study, the main aim of which was to provide evidence that could allow planning of a further confirmatory study, we focused on the efficacy of the training on gait performance. Therefore, the sample size was determined without any *a priori* formal statistical hypothesis. Clinical-demographic characteristics are detailed in Table 1. The local Ethics committee approved the study and each patient gave his/her written informed consent to study participation and data publication.

**Table 1:** Clinical demographic data.

	**Gender**	**Age (yy)**	**dd (yy)**	**H&Y stage**	**LEDD mg/day**	**MMSE**	**DBS setup (left/right electrode)**					**UPDRS-OFF**		**UPDRS-ON**		**TUG (s)**		**10 MWT (m/s)**		**BBS**		**FES**		**ACE–R**	
							**Implantation (mm)**	**Stimulation frequency (Hz)**	**Active contacts**	**Impulse amplitude (V)**	**Impulse width (μs)**	**Pre**	**Post**	**Pre**	**Post**	**Pre**	**Post**	**Pre**	**Post**	**Pre**	**Post**	**Pre**	**Post**	**Pre**	**Pre**
DBS (*n* = 10)	M	57	16	2.5	900	25	36	200/200	1–0/6–7–	3.6/3	60/60	20	11	11	9	11	9	0.8	1	39	54	54	51	79	91
	F	67	14	3	700	27	14	240/240	1–3–/5–7–	1.9/2.2	120/120	22	14	14	10	25	6	1	1.5	37	53	46	35	83	88
	M	64	17	2.5	750	26	22	200/200	2+0/4–7–	2.3/2	60/60	35	17	17	11	10	6	1.1	1.4	37	51	40	32	84	93
	M	56	16	3	750	26	18	200/200	3–/7–	3.6/3.2	60/60	48	24	24	19	16	7	1	1.4	50	54	34	28	80	85
	F	58	15	3	650	27	17	210/210	1–2–/4–6–	2/2.5	60/60	47	32	42	16	17	13	1	1.5	42	49	27	21	80	87
	F	66	17	3	600	26	24	200/200	3–2–/6–7–	3.1/3.6	60/60	28	14	14	10	16	12	1.3	1.4	41	44	34	20	83	83
	M	66	10	2.5	700	27	22	130/130	1+/5–	3.6/3.2	60/60	26	12	12	9	20	13	1.3	1.5	42	43	27	27	79	88
	F	66	18	2.5	900	25	16	240/240	2–0/6–7–	2.8/3	60/90	32	18	18	10	12	9	0.9	1.2	50	52	26	16	77	79
	M	56	11	3	950	25	18	210/210	3–1–/4–5–	2.8/2.8	60/60	47	17	17	10	16	8	1.3	1.7	37	52	36	26	75	79
	M	67	17	3	500	26	15	210/210	2–1–/4–7–	2.2/2.6	60/60	32	22	22	18	18	15	0.8	1.1	47	54	39	29	74	87
	6M 4F	62 ± 5	15 ± 2	3 (2.5–3)	740 ± 143	26 (25–27)						32 (27–44)	17 (14–21)	17 (14–21)	10 (10–15)	16 ± 4	10 ± 3	1 ± 0.2	1.4 ± 0.2	42 (38–46)	52 (50–54)	35 (29–40)	28 (22–31)	80 (77–82)	87 (83–88)
Within-group difference													<0.001		0.01		0.003		<0.001		0.002		<0.001		0.001
Non–DBS (*n* = 10)	F	65	11	2.5	350	27						46	35	26	23	46	33	0.8	0.9	30	33	40	22	87	88
	M	58	12	2.5	700	27						34	27	26	24	24	20	1.5	1.7	47	48	37	36	83	86
	F	57	11	2.5	700	27						38	28	30	16	20	18	1.3	1.4	42	53	38	28	83	88
	F	64	12	3	800	27						50	39	27	24	22	19	0.7	0.7	39	47	36	30	88	88
	M	65	15	2.5	750	25						34	25	28	22	24	21	0.5	0.5	36	41	31	28	79	80
	M	65	13	3	700	27						35	25	27	26	26	25	1.6	1.9	43	53	40	20	87	88
	F	67	15	2.5	900	27						32	24	28	22	18	17	1	1.1	40	48	36	33	80	86
	M	64	16	2.5	675	25						50	38	27	19	13	11	1.3	1.4	44	44	39	35	80	89
	F	58	14	3	750	27						34	25	28	26	14	14	1.1	1.2	46	46	33	30	85	86
	M	60	17	2.5	700	27						40	30	28	24	15	13	1.3	1.4	44	49	42	23	76	88
	5M 5F	62 ± 4	14 ± 2	2.5 (2.5–2.9)	703 ± 141	27 (27)						37 (34–45)	28 (25–33)	28 (27,28)	24 (22–24)	22 ± 9	19 ± 6	1.1 ± 0.4	1.2 ± 0.4	46 (39–44)	48 (44–49)	38 (36–40)	29 (24–32)	83 (80–87)	88 (86–88)
Within-group difference													<0.001		0.003		0.03		0.005		0.003		0.005		0.01
Baseline between-group difference	0.7	0.9	0.2	0.2	0.6	0.1						0.2		0.009		0.1		0.5		0.6		0.8		0.1	
Pre-post between-group difference													<0.001		0.02		0.001		<0.001		0.2		0.9		0.2

*Mean values refer to mean ± sd or median (iqr). Post hoc t-test p-values are reported. Dd, disease duration. For left electrode, 0 was the most ventral contact and 3 was the most dorsal; for the right electrode, 4 was the most ventral contact and 7 the most dorsal*.

### Gait Training

Patients underwent a RAS-assisted treadmill training within a conventional rehabilitation program. Patients practiced one session of RAS-assisted treadmill training and one session of conventional physiotherapy once a day, 6 days a week, for 1 month.

RAS-assisted treadmill training was conducted using the Gait Trainer 3 (GT3) (Biodex; Shirley, NY, US). GT3 is a validated electronic walkway equipped with an instrumented deck that monitors and records step length, step speed and step symmetry to train neurologic patients (including PD and stroke) in achieving better gait performance. By combining audio-visual feedback and music-assisted therapy, GT3 helps promoting neuroplasticity-based recovery processes (18, 29). The device provides clinicians with data on load distribution during the stance phase, the actual location of feet, and the matching of the length of the step performed with a pre-determined step length at a certain speed (audiomotor synchronization).

Each GT3 session lasted 30 min, during which the patients were provided with simple, two–accent, metronome sounds (70 dB_SPL_) to which they were instructed to synchronize the footsteps while walking. The bpm of the soundtrack (animals everywhere) was calculated considering patients' cadence measured at the beginning of the rehabilitation program at a comfortable gait speed (mean ± sd 85 ± 5 bpm, about 0.43 m/s) and was then increased of 5 bpm every 3 min of walking up to 120 bpm (about 0.61 m/s) or the maximum tolerable bpm. When the patients achieved the maximum tolerable or the target bpm, the 30 min session began. This procedure was repeated every day of treatment. We adopted the 120-bpm target frequency as it is intermediate between those potentially worsening step length and gait cadence, especially when too low (i.e., 60–90 bpm) or too high (>150 bpm) (30). The patients achieved the target bpm of 120 within the third-fourth session. The bpm value was kept if the patient was able to maintain step length symmetry safely; otherwise, the training was conducted using the same velocity as in the previous session. The interval between one beat/step and the subsequent one was kept constant in each session. The progression of intensity of training was individually adapted in order to prevent fatigue, for which patients were carefully monitored; in the case, walking speed was reduced to a comfortable pace (mean ± sd 0.52 ± 0.1 m/s). Furthermore, heart rate and pulse oximetry were monitored during each session. During the training, the patient was required to maintain step length symmetry as displayed on a screen put in front of the patient and to refrain from holding onto the handrails of the treadmill as much as possible.

Each session of conventional physiotherapy lasted 60 min. It consisted of exercises aimed at targeting flexibility, balance, gait, and muscular tone and resistance (31, 32). Orthoticism and body alignment were monitored thoroughly.

The two session were separated by at least an hour of break. The order of session was random but counterbalanced within each patient and group. LEDD and DBS setup were kept constant during the monthly rehab training.

### Outcome Measures

Patients were evaluated before and after training completion using the Unified Parkinson's disease rating scale (UPDRS) part 3 in OFF–dopa and ON–dopa, the Falls efficacy scale (FES), the Berg Balance Scale (BBS), the Timed Up&Go test (TUG), the 10–meter walking test (10 MWT), and the Addenbrooke's Cognitive Examination–Revised (ACE–R). Furthermore, once the patient achieved his/her target bpm (third-fourth session), he/she was provided with EEG recording while walking on the RAS-treadmill. EEG was also recorded at the last day of the training.

### EEG Recording and Analysis

EEG was recorded about 10 min after the patient started walking on the GT3, for 10 min. All patients were in ON–state. The participant wore a standard 19–electrode headset wired to a Brain–Quick System (Micromed; Mogliano Veneto, Italy). Patients were prohibited from drinking coffee, smoking, and changing their bedtime during the 3 days prior to EEG recording. This was easily checked, as the participants were in–patients.

EEG was sampled at 512 Hz, filtered at 1–45 Hz, referenced to both the mastoids, and notch–filtered. Impedances were constantly monitored to be <5 kΩ. An electrooculogram (EOG) with a bipolar montage was also collected. Data were pre–processed using EEGLab. EEG recordings were first visually inspected to identify and remove data affected by prominent artifacts across all the recording channels. Then, the data decomposed into neural and artifactual components using the Infomax algorithm Independent Component Analysis (ICA) (33). It has been reported that employing ICA and linear autoregressive model easily allow identifying the periodical motion artifacts, as periodic was the walking activity in our study, present in the EEG recordings (34–36). Continuous data were then segmented into epochs starting from the left heel strike (HS) and ending at the next one to capture a complete stride. EEG segmentation was based on data synchronized from the important time points (left and right HS, left and right toe off—TO) furnished by the wireless G–Sensor inertial sensor (BTS Bioengineering; Milan, Italy) and used to extrapolate gait epochs. Thus, the single trial spectrograms were time–warped using a linear interpolation function, with the gait data used as milestones for realigning the EEG signals' time axes (i.e., aligning the time–points of the epochs for the left HS, right TO, and the next left HS, which were time–warped to 0, 50, and 100% of the gait cycle, respectively) (34, 35, 37–39). Thus, left HS served as reference point in the gait cycle to which all segments were aligned. We thus obtained 356 ± 32 epochs after bad epoch removal (visual inspection and ICA).

We estimated the Event–Related Spectral Perturbation (ERSP) in relation to RAS provision in the alpha and beta frequency ranges (given that their spectral power changes are the most significantly occurring during treadmill walking) and in three regions of interest (ROI) related to motor control function (sensorimotor affordance, P3/4 and T3/4; motor execution, C3/4, Cz, and FCz; and motor planning, F3/4, Fz, and FCz), with visual region serving as a reference (O1/2) (34, 35, 40). Overall, such ROIs are of particular relevance to interval timing, rhythm perception, and auditory–motor coordination (20, 41–43). Specifically, we performed a time–frequency analysis (TFA) related to the phases of the gait cycle, so to assess changes in spectral measures within the gait cycle and between hemispheres (whether we found a ROI as significant) (44, 45). Thus, TFA was performed in 1 Hz step size on the entire frequency range using a “Hanning” taper, for each ROI (and side within the ROI). Using a sliding–window approach, the taper (length 100 ms) was moved along the epochs in 5 ms steps. Such a taper setting is best for the assessed frequencies. Fourier transformations were performed on the single trials prior to averaging. The so–obtained power estimates were baseline corrected (we used the mean value across the whole epoch) for each frequency to obtain the relative signal change.

### Statistical Analysis

Clinical changes were estimated by using Friedman or repeated measure ANOVA where appropriate, with the factor time (two levels: PRE and POST), and *group* (two levels: DBS and non–DBS). Bonferroni corrected *post–hoc t–*tests were carried out (α = 0.05, *p-*value = 0.05/n comparisons). With regard to the EEG data analysis, ANOVA analysis was conducted with the factors *time*–*window* within the gait cycle (six levels: LTO⌢ 25% of gait cycle, 25% of gait cycle⌢ LHS, LHS⌢ RTO, RTO⌢ 75% of gait cycle, 75% of gait cycle⌢ RHS, and RHS⌢ LTO), *group* (two levels: DBS and non–DBS), *ROI* (four levels: visual, sensorimotor, motor execution, and motor planning), *frequency*–*range* (two levels: alpha and beta), and *time* (two levels: PRE and POST). The false discovery rate was controlled using the Benjamini–Hochberg procedure (46, 47). Statistical significance for main effects was set at 0.05, and followed by *post–hoc* comparisons (with Fisher's LSD correction). Statistical analysis was conducted according to the intention-to-treat principle using multiple imputations to account for missing data (48). The personnel who conducted the statistical analysis was blinded to patient allocation.

## Results

### Baseline

The DBS group consisted of 10 patients who were implanted with DBS about 1–3 years prior to study inclusion (Table 1). DBS setup (electrodes and Kinetra stimulators from Medtronic; Minneapolis, MN, USA) was characterized by a stimulation frequency of 130–240 Hz, an impulse amplitude of 2.2–4.9 V, and an impulse width of 60–120 μs (Table 1). The non–DBS group (control group) consisted of 10 patients matched for age, gender, disease duration, H&Y stage, LEDD, and MMSE. There were not significant outcome differences at baseline between the groups (all *p* > 0.1) but UPDRS–ON (Table 1). Clinically, all patients complained of mild to moderate disability and impaired postural reflexes (H&Y). Motor signs were mild-to-moderate in frequency and intensity, sufficient to affect, but not to prevent, a function (UPDRS). Patients were somewhat or fairly concerned about falling (FES), showed a mild-to-moderate impairment in performing static and dynamic activities (BBS and TUG), and complained of a very mild cognitive decline (ACE–R). Gait speed (10 MWT) was intermediate in the commonly reported range in PD patients, but it was however lower than the gait speed for healthy elderly (around the 60th centile), consistently with the decrease in BBS and increase in TUG.

**Figure 1:**
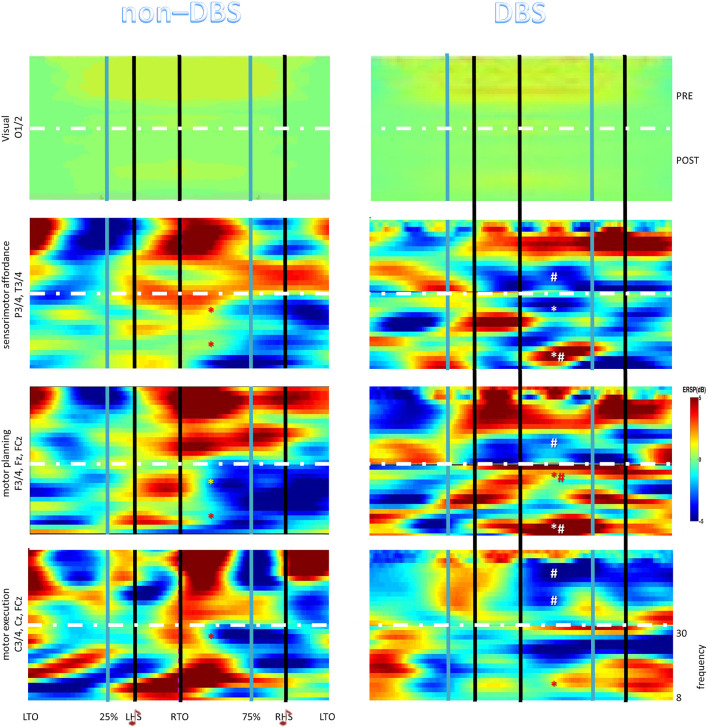
The Event–Related Spectral Perturbation (ERSP) plots relative to the full gait cycle showing the average changes in spectral power during the gait cycle for the different electrode groups (brain areas). The horizontal axis is the percentage of gait cycle (as we performed a time–warping analysis) referred to the heel strikes (HS) and the toe offs (TO). *denotes post-pre significant changes (*p* < 0.001), #between-group post-pre significant differences (*p* < 0.001).

The TFA disclosed significant and sharp intra-stride changes in spectral power and a clear distinction between alpha and beta power modulation in the DBS group. Specifically, we found a beta power increase and alpha power decrease within the left/right sensorimotor and the left/right motor execution ROIs and an alpha/beta power decrease in the left/right motor planning ROI in the [25% of gait cycle⌢ LHS⌢ RTO⌢ 75% of gait cycle] time–window, with particular regard to the end of each stance phase (i.e., when the leading foot was in HS and the trailing foot was in TO) (Figure 1). Therefore, we found that power changes occurred in the sensorimotor, motor execution, and motor planning areas more for the contralateral TO/ipsilateral HS than for the ipsilateral TO/contralateral HS. Consequently, there were no significant left/right ROI differences in the overall sequencing of strides, as each activation phase was counterbalanced between the hemispheres within each, single phase of the gait cycle.

Conversely, we found a single response in the non–DBS group, i.e., an alpha and, even more, beta power increase in the [25% of gait cycle⌢LHS⌢RTO⌢75% of gait cycle] time–window within each area but the visual one, which was followed by a short beta power decrease only within motor planning ROI (Figure 1). Left/right ROI activations were overall counterbalanced in the sequencing of strides within each, single phase of the gait cycle.

### Post–training

All enrolled patients completed the monthly rehab session, without any adverse event. After the training, the DBS group improved more than non–DBS group did in: (i) 10 MWT, where DBS patients achieved a greater substantial meaningful change (at least 0.1 m/s) than non-DBS patients did (*time* × *group F* = 18, *p* = 0.005, η^2^ = 0.658; DBS group *F* = 28, *p* < 0.001, η^2^ = 0.847; non–DBS group F = 25, *p* < 0.001, η^2^ = 0.833); (ii) TUG, which improved above the minimal detectable (MDC) change only in the DBS group (*time* × *group* F = 15, *p* = 0.001, η^2^ = 0.625; DBS group F = 22, *p* = 0.001, η^2^ = 0.818; non–DBS group F = 6.6, *p* = 0.03, η^2^ = 0.64); and (iii) UPDRS–OFF, as DBS patients showed slight motor signs compared to non-DBS patients, who showed slight-to-mild motor signs (*time* × *group* F = 25, *p* < 0.001, η^2^ = 0.714; DBS group F = 26, *p* < 0.001, η^2^ = 0.838; non–DBS group F = 25, *p* < 0.001, η^2^ = 0.833). *Post–hoc* comparisons are reported in Table 1.

Conversely, both groups equally improved in BBS (both groups above the MDC; *time* × *group p* = 0.6; DBS group F = 16, *p* = 0.003, η^2^ = 0.775; non–DBS group F = 18, *p* = 0.002, η^2^ = 0.791), FES (both groups from moderate to low concern about falls; *time* × *group p* = 0.9; DBS group F = 28, *p* < 0.001, η^2^ = 0.847; non–DBS group F = 14, *p* = 0.005, η^2^ = 0.756), and ACE–R (*time* × *group p* = 0.2; DBS group F = 23, *p* = 0.001, η^2^ = 0.823; non–DBS group F = 10, *p* = 0.01, η^2^ = 0.705). *Post hoc* comparisons are reported in Table 1.

The EEG pattern was significantly modified after the training in both groups and frequency–ranges, but with specific differences concerning the time–windows within the gait cycle and the ROIs (Figure 1) (*group* × *time* × *frequency–band* × *ROI* × *time–window* interaction F = 3.5, *p* < 0.001, η^2^ = 0.999).

Specifically, the baseline ERSP pattern in the DBS group was replaced by an alpha and beta power decrease in the [25% of gait cycle LHS RTO 75%] time–window within the sensorimotor and motor execution ROIs and by a beta decrease and alpha increase in the abovementioned time–window within the motor planning ROI (Figure 1). Power changes occurred in a ROI more for the contralateral TO/ipsilateral HS than for the ipsilateral TO/contralateral HS, as observed in the baseline recording. Consequently, there were no significant left/right ROI differences in the overall sequencing of strides.

The ERSP scenario was significantly different in the non–DBS group (*group* × *time* × *frequency–band* × *ROI* interaction F = 4.6, *p* = 0.004, η^2^ = 0.899), where the ERSP baseline pattern was replaced by a beta power decrease and an alpha power increase within each ROI in the abovementioned time–window (*time* × *frequency–band* interaction F = 25, *p* < 0.001, η^2^ = 0.999; *time* × *ROI* interaction F = 12, *p* < 0.001, η^2^ = 0.999) (Figure 1). Within- and between-group *post-hoc* comparisons were all *p* < 0.001 (Figure 1).

**Figure 2:**
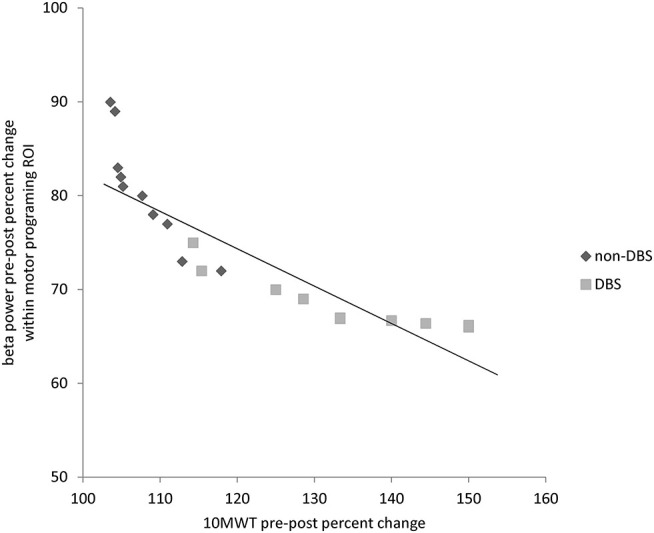
Correlation plot between beta power pre-post percent changes within motor programming ROI and pre-post percent improvement in 10-meter walking test (10 MWT).

With regard to clinical and electrophysiological correlations, we found a significant, negative correlation between beta power percent change (decrease) within motor programing ROI and 10 MWT percent change (increase) (*r* = −0.772, *p* < 0.001) (Figure 2).

## Discussion

To the best of our knowledge, this is the first time that patients with PD using DBS were provided with gait training with RAS-aided treadmill gait training in add-on to conventional training and were investigated for the dynamics of EEG responses in relation to the gait cycle to such treatment. Our work presents two main findings. First, RAS-assisted gait training plus conventional physiotherapy allowed the patients with DBS to achieve a greater improvement in gait velocity (as per 10 MWT score), overall motor performance (as per UPDR–III), and timed velocity and self-confidence in balance, sit-to-stand (and vice versa) and walking (as per TUG), as compared to patients without DBS. Furthermore, the coupled training enabled all patients to improve in the ability to safely balance during a series of predetermined tasks (dynamic and static balance) (as per BBS scores), the overall cognitive performance (including attention/orientation, memory, language, verbal fluency, and visuospatial skills; as per ACE–R), and the fear of falling (as per FES). Second, the main mechanism subtending the greater improvement in DBS patients compared to non–DBS ones may be due to the remodulation of the sensorimotor beta oscillations along the gait cycle induced by the functional association between physiotherapy plus RAS-treadmill and DBS.

### Clinical Aftereffects

The clinical aftereffects our patients reported are consistent with previous data on the efficacy of RAS-assisted gait training plus conventional physiotherapy in improving gait performance in patients with PD (15, 16, 18, 49). Actually, our rehab paradigm improved gait velocity, stability and overall mobility, and reduced the risk for falls and, thus, disability, in PD patients, in whom changes in gait often affect disability, morbidity, and mortality (50, 51). Specifically, patients achieved to walk faster, take longer strides, spend less time in stance and double support, and be affected by a lower gait variability and asymmetry and a less impaired postural control. All such findings are consistent with those coming from treadmill training (including gait speed, step length, stride variability, balance, and freezing) (15, 16, 52–56).

The specific efficacy of treadmill-assisted gait training is likely to depend on the higher intensity, repeatability, and controllability of gait under treadmill gait therapy as compared to overground gait therapy. Noteworthy, all such issues are cardinal for an effective rehabilitation of gait in patients with PD (57, 58). In detail, treadmill-assisted gait training provides patients with continuous sensory stimulation, external sensory cues, activation of gait central-pattern-generator circuits, visual feedback, and motor learning (59, 60). Furthermore, treadmill-assisted gait training ensures a safer and easier lower limb mobility, higher trunk control, larger range of movements of lower limbs joints, and a more controlled cardiorespiratory demand (15, 16).

All these effects were found in the DBS group using RAS. Therefore, treadmill-assisted gait training, coupled to RAS, could be helpful to maintain the clinical gait improvement after DBS surgery. Even though this issue deserves further studies (14, 20), we formerly found that RAS-assisted treadmill gait training was superior to stand-alone conventional treadmill gait training in improving motor performance in patients with PD (18). In the present study, patients with DBS showed gait-related functional gains, as those previously observed in patients without DBS. Furthermore, some specific, higher benefits in gait performance were appreciable in patients with DBS, as compared to those without DBS. Thus, our data extend the conventional physiotherapy and RAS-assisted gait training benefits to patients with DBS, who have not been assessed specifically for RAS-assisted gait training aftereffects yet (14, 20, 21). Despite this is a case-controlled pilot study, the clinical aftereffects of conventional and RAS-assisted gait training are of importance, given that DBS has shown limited efficacy in gait abnormalities (17, 61), and that the motoric aftereffects of DBS require specific motor rehabilitation to maintain and improve the acquired functional gains (12).

The clinical benefits of conventional physiotherapy and RAS-assisted gait training are likely to depend on the intensity (every a day, six time a week, for 1 month) and session-by-session repeatability of motor training. The RAS-assisted gait training as an add-on treatment ensured patients with precise rhythmicity of walking and high gait cycle repeatability within and along rehab sessions, which were also task–oriented (RAS–step synchronization and audio–motor feedback) (18, 49). Actually, all such issues are cardinal for an effective rehabilitation of gait in patients with PD (57, 58).

One may argue that the great effects of treadmill walking and conventional physical therapy (excluding RAS effects) on the outcome measures was foreseeable, given that both patient groups were treated with an equal dosage of conventional and RAS-assisted gait training and were included in the study according to the absence of any physical therapy and treadmill walking in the 6 months prior to study onset. However, DBS patients showed some further positive effects as compared to non-DBS patients. This may depend on combined effects of DBS, physiotherapy and RAS-treadmill on neural oscillatory mechanisms related to gait cycle phases as suggested by the EEG data analyses.

### Putative Model of Interaction Between RAS-Plus-Physiotherapy and DBS

The temporally predictable cues can support gait initiation and continuation both immediately and long after an extensive period of RAS by resetting the PD–related variability of sensorimotor timing skills (19, 55, 62, 63). In particular, RAS training improves patent's capacity to coordinate steps to the timing and rate of RAS, and to the time goal–directed movements to the beat onsets, with overall positive effects on gait performance (18). The exact neurophysiological underpinnings of RAS have to be elucidated clearly. Auditory-motor entrainment and the modulation of corticostriatal activity are considered the main mediators of the effects of the RAS (64). Specifically, RAS has been reported to favor the recovery of auditory–motor coupling mechanism within a large network (including both subcortical and cortical regions such as the cerebellum, supplementary motor area, and premotor cortex) in patients with PD (65–67). Auditory–motor coupling is important for internal timing and sensorimotor coordination mechanisms related to repetitive, semi–automated movements, including gait and upper limb pendolarity (49). These functions are impaired in patients with PD owing to an altered, dopamine–dependent, beta–band activity within basal ganglia–thalamo–cortical circuitry, with particular regard to STN and SMA, which are critical for externally–paced auditory–motor entrainment (26, 41, 68, 69). RAS has been show to be capable to remodulate such deteriorated beta band networking (18, 64, 70, 71), thus being the most important contributor to clinical improvement, similarly to what occurs using intensive gait training, levodopa and DBS (20, 60, 72, 73). Particularly, gait training has been shown to remodulate beta oscillations in a task-dependent manner, with consequential positive effects on gait stability, speed, and step length control, gait adaptation and anticipatory postural adjustments, visuomotor integration (71). With this regard, it could be that the beta band may subtend the temporal coordination of sensorimotor processes, and hence be involved in the temporal coordination of the HS during walking (74).

Actually, beta activity is hypersynchronized within basal ganglia–thalamo–cortical circuitry, resulting in an impairment of movements related to deficient timekeeping functions and dynamic scaling and sequencing of complex sensorimotor processes, including gait (26, 75–78). The correlation analyses in our work consistently support this issue, in that the beta modulation within frontal ROI was correlated with gait performance improvement significantly. Furthermore, it is worth noting that gait training acts on such deteriorated networks as well as levodopa and DBS do, improving audio–motor connectivity (79–81). Therefore, the hypothesis that physiotherapy plus RAS interacted with DBS synergistically by means of beta modulation, eventually improving gait performance seems reliable. In this regard, the synergy between RAS, physiotherapy and DBS is suggested by the specific post–treatment differences we found between DBS and non–DBS gait training outcomes concerning beta oscillations.

At baseline, DBS patients showed a better beta modulation pattern along the gait cycle, as compared to non–DBS patients. This regarded the frontal electrodes in particular, i.e., a beta power peak during the double support phase, which is in keeping with a feedback signaling of step synchronization correctness (82–84). Actually, the non–DBS patients showed a beta oscillatory activity increase externally to the double support and a lack of beta suppression during contralateral foot lift, which is in keeping with the detrimental beta oscillatory activity along the basal ganglia–thalamo–cortical circuitry in patients with PD (23, 50, 60, 71, 72, 85–90). This condition reflects the restriction of patients with PD into timing function–based motor tasks and the hastening phenomenon (i.e., the involuntarily acceleration of motion instead of precisely synchronizing with rhythmic external cues) (24, 91–93).

The baseline alpha and beta pattern was remodulated at the end of the training largely in both groups. However, specific between–group differences were appreciable. In particular, gait training in non–DBS patients favored a gait cycle phase specific, alpha and beta power modulation within each sensorimotor area, as per our previous findings (18). Therefore, RAS plus physiotherapy may have reshaped the intrinsic rhythmicity related to gait cycle, thus improving motor performance and positively affecting bradykinesia, which is properly related to beta modulation (23, 26, 71, 72, 85, 87, 94). However, such a beta oscillation modulation has been also reported during walking, cycling, stepping training and, noteworthy, DBS and levodopa treatment (20, 60, 71–74, 86, 90). On the other hand, the association between DBS and RAS plus physiotherapy may have allowed a greater focusing on beta frequencies, mainly in the double stance phase and within sensorimotor and frontal electrodes, in keeping with the beta desynchronization during movement execution followed by a beta rebound between two consecutive rhythmic movements (95, 96). In other words, DBS could have focused and boosted RAS plus physiotherapy aftereffects mainly acting on the feedback signaling of step synchronization correctness during the gait training (as indicated by the modulation of beta activity during the double stance phase) within parts of the wide auditory–motor coordination network entrained by RAS (including SMA and STN) and of the sensorimotor network entrained by gait practice on the treadmill itself (including motor and premotor cortices, primary sensorimotor cortex, and dorsal anterior cingulate cortex) (97). This coupled effect between RAS plus physiotherapy and DBS is suggested by the significant beta modulation we found within the frontal (motor program) ROI, that includes SMA, which is highly connected with STN and is critical for beta–band based internal synchronization mechanisms, including gait (26, 41, 41, 42, 68, 69, 98, 99). In this regard, the application of transcranial magnetic stimulation over the SMA has been shown to transiently reduce beta oscillations in the STN as measured with DBS electrodes (100). Furthermore, the focusing of beta activity modulation in this part of the gait cycle and in SMA electrodes suggest an error correction in the next temporal parameters of step cycles, i.e., a retrospective evaluation of gait performance so as to update and improve the subsequent gait performance (84).

All the above mentioned issues suggest that the oscillatory effects we observed are likely to depend on the improvement of the basal ganglia–thalamo–cortical circuitry induced by the synergy between RAS plus physiotherapy and DBS. The former activates gait-related sensorimotor and auditory–motor coordination network at cortical level (through basal ganglia–thalamo–cortical circuitry that plays a critical role in modulating beta band activity during synchronization tasks (i.e., RAS treadmill) (42, 43, 93, 97, 98, 101, 102). The association between audiomotor synchronization and the amount of sensorimotor information related to gait execution, together with DBS stimulation, may modulate beta oscillations within basal ganglia–thalamo–cortical circuits that contribute to long–term potentiation(LTP)–like and spike–timing dependent plasticity mechanisms at the cortical level (maybe through GABAergic and cholinergic neural transmission) (103–111). Actually, we can hypothesize that DBS and RAS plus physiotherapy may interact through a sort of associative plasticity, which is already triggered by the coupling of RAS with steps during gait training (18, 49), thus further suggesting that there may be a synergy among DBS, RAS, and physiotherapy. Indeed, the repeated stimulation of basal ganglia–thalamo–cortical loop by part of DBS and RAS plus physiotherapy may have led to a LTP–like plasticity potentiation in the motor cortex, of which beta modulation is a feature (112, 113). Overall, the issues we described suggest that the reported EEG activities likely represent a true synchronization of three interacting oscillators, i.e., DBS, RAS, and physiotherapy, with significant consequences on beta–frequency based cortical excitability.

### Limitations

There are some limitations to acknowledge. First, the small sample enrolled and the relatively high standard deviation values in some post-treatment measures may have limited the significance of some outcome measure changes. However, this was a pilot, case–control study aimed at preliminary assessing the effectiveness of RAS training in patients with PD using DBS.

Second, there was not a control experimental group of PD patients undergoing treadmill-based rehabilitation without RAS. However, we have formerly shown that treadmill-based rehabilitation without RAS provides patients with inferior gait outcomes than treadmill-based rehabilitation with RAS (18). Nonetheless, further clinical trials on patients who did not practice RAS, practiced different RAS frequencies, or withdrew DBS, are necessary to confirm our promising findings (114, 115).

Third, one may be concerned on the fact that surface EEG recordings from the frontal ROIs do not selectively detect the oscillatory activity of the STN and SMA, which are known to be crucial for the externally–paced auditory-motor entrainment. However, it has been reported that the neural dynamics among STN, SMA, premotor cortices (midline electrodes) and prefrontal cortices (frontal electrodes) can be deduced by surface EEG (116–120). Nonetheless, a key confirmation would arise from the recording of surface EEG using higher electrode density and of the oscillation dynamics in basal ganglia through DBS electrodes (bilateral STN) in PD patients during RAS-based training.

Fourth, there could be a concern on EEG recording while using DBS and on the frequency bands we analyzed, not including the gamma frequency range. Indeed, we did not find focally distorted spreads of EEG signals compatible with surgery–related skull defects or DBS stimulation. Even though gamma oscillatory activity is also relevant in patients with PD (for instance, the swing phase is accompanied by gamma power decrease in the contralateral central electrodes, beside a significant bilateral alpha-beta power decrease) (117, 121–123), gamma recording is easily affected by movement artifacts and may need specific equipment to reliably reported and discussed (18). However, this interesting aspect may be addressed in future, larger clinical trials.

Last, one may have expected a larger effect on UPDRS–III than the one we found. This is likely to depend on the fact that we enrolled patients with long–term carry–over effects of chronic, high–frequency DBS (118, 124–126).

## Conclusions

Our work suggests that RAS-assisted gait training coupled to conventional physiotherapy is an effective tool to improve gait performance in patients with PD with and without DBS. Interestingly, patients with DBS achieved better results concerning gait velocity and stability. Specifically, patients with DBS may have benefited more from this approach compared to those without DBS owing to a more focused and dynamic re–configuration of sensorimotor beta oscillations related to gait secondary to DBS effects on RAS gait training plus conventional physiotherapy. Potentiating DBS using RAS gait training plus conventional physiotherapy may help restoring a residually altered beta–band response profile despite DBS intervention, thus better tailoring the gait rehabilitation of PD patients submitted to DBS.

## Data Availability Statement

The datasets generated for this study are available on request to the corresponding author.

## Ethics Statement

The studies involving human participants were reviewed and approved by IRCCS Centro Neurolesi Bonino Pulejo. The patients/participants provided their written informed consent to participate in this study.

## Author Contributions

RC: conceptualization and reviewing and editing. AN and LP: methodology. AN and LB: investigation. CS, DL, LB, and AM: data curation. AN: original draft preparation. RC and DB: supervision. All authors contributed to the article and approved the submitted version.

## Conflict of Interest

The authors declare that the research was conducted in the absence of any commercial or financial relationships that could be construed as a potential conflict of interest.
